# FASTER: an unsupervised fully automated sleep staging method for mice

**DOI:** 10.1111/gtc.12053

**Published:** 2013-04-28

**Authors:** Genshiro A Sunagawa, Hiroyoshi Séi, Shigeki Shimba, Yoshihiro Urade, Hiroki R Ueda

**Affiliations:** 1 Laboratory for Systems Biology, RIKEN Center for Developmental BiologyKobe, 650-0047, Japan; 2 Department of Integrative Physiology, Institute of Health Biosciences, The University of Tokushima Graduate SchoolTokushima, 770-8503, Japan; 3 Department of Health Science, College of Pharmacy, Nihon UniversityFunabashi, 274-8555, Japan; 4 Department of Molecular Behavioral Biology, Osaka Bioscience InstituteSuita, 565-0874, Japan; 5 Laboratory for Synthetic Biology, RIKEN Quantitative Biology CenterKobe, 650-0047, Japan; 6 Department of Biological Sciences, Graduate School of Science, Osaka UniversityToyonaka, 560-0043, Japan; 7 Graduate School of Frontier Biosciences, Osaka UniversitySuita, 565-0871, Japan; 8 Department of Systems Pharmacology, Graduate School of Medicine, University of TokyoTokyo, 113-0033, Japan

## Abstract

Identifying the stages of sleep, or sleep staging, is an unavoidable step in sleep research and typically requires visual inspection of electroencephalography (EEG) and electromyography (EMG) data. Currently, scoring is slow, biased and prone to error by humans and thus is the most important bottleneck for large-scale sleep research in animals. We have developed an unsupervised, fully automated sleep staging method for mice that allows less subjective and high-throughput evaluation of sleep. Fully Automated Sleep sTaging method via EEG/EMG Recordings (FASTER) is based on nonparametric density estimation clustering of comprehensive EEG/EMG power spectra. FASTER can accurately identify sleep patterns in mice that have been perturbed by drugs or by genetic modification of a clock gene. The overall accuracy is over 90% in every group. 24-h data are staged by a laptop computer in 10 min, which is faster than an experienced human rater. Dramatically improving the sleep staging process in both quality and throughput FASTER will open the door to quantitative and comprehensive animal sleep research.

## Introduction

**Figure 1:**
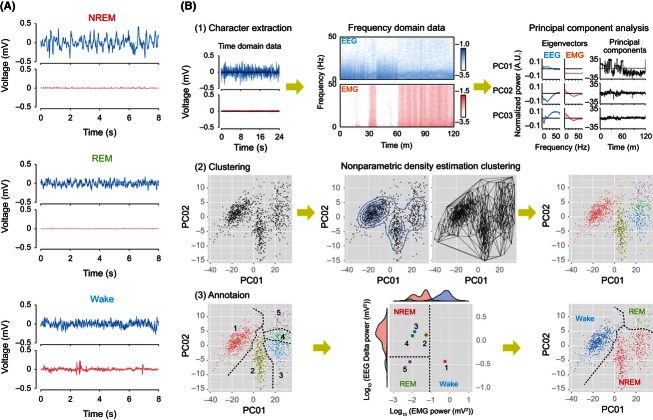
(A) Representative EEG and EMG time-domain data during 8 s of NREM sleep, REM sleep and wake status. NREM sleep is characterized by high-amplitude EEG delta waves (0.5–4 Hz) with low EMG power, REM sleep is characterized by low-amplitude, high-frequency EEG theta waves (6–10 Hz) with very low EMG power, and wake is characterized by high and varying EMG power. (B) An overview of FASTER, the unsupervised fully automated sleep staging. (1) Each epoch's power spectrum of both the EEG and EMG is calculated by FFT. EEG/EMG power spectra are transformed into values of principal components by principle component analysis (PCA), and the values of top four principle components are used for further analysis. (2) Epochs are clustered by the nonparametric density estimation clustering using the principal components. This clustering method estimates the probability density of the dataset and defines a cluster as a high-density area that is connected by Delaunay triangulation. The number of the clusters is automatically calculated by the method. (3) The clusters are annotated according to the median logarithm of EMG power and EEG delta power of each cluster (middle panel). Histograms around the middle panel represent the relative counts of each sleep/wake stage by the logarithm of EMG power (upper histogram in the middle panel) and the logarithm of EEG delta power (left histogram in the middle panel). In this representative case, cluster 1 is first annotated as ‘wake’ by the median logarithm of EMG power. Then, the remaining clusters are annotated as ‘NREM sleep’ and ‘REM sleep’ by the median logarithm of EEG delta power. The final staging is shown in the right panel.

Ever since the discovery of sleep/wake status relationship to electroencephalography (EEG) in the early 20th century, sleep staging based on EEG has been the standard method to evaluate sleep/wake status in animals. In mice, states of consciousness are classified into at least three stages: nonrapid eye movement sleep (NREM sleep), rapid eye movement sleep (REM sleep) and wake. Determination of an animal's sleep/wake stage is based on visual inspection of EEG and electromyography (EMG) of the animal by well-trained human raters with some or no computational support. The classical sleep scoring criteria for mice use the amplitude of selected frequency bands of EEG and EMG (Fig. 1A). NREM sleep is characterized by large and slow EEG waves with low EMG amplitude, REM sleep has lower and faster EEG with very low EMG amplitude, and wake has an EEG pattern similar to REM sleep with very high amplitude of EMG.

There are two problems scoring sleep/wake stages by visual inspection: quality control and throughput. Scoring sleep stages depends on human rater bias and will differ between two human raters (inter-rater variance) and a human rater analyzing the same data multiple times (intrarater variance). The main reason why visual inspection by a human rater is still used for sleep staging is related to the fundamental difficulty of sleep staging in drawing boundaries between different stages. There are many factors causing this difficulty, such as the differences between individual animals, the noise of EEG/EMG recording device and variance in surgical techniques used in electrode implantation that can make the definition of rigid boundaries problematic. Visual inspection aims to compensate for these sources of variance in the EEG/EMG data, sacrificing objectivity in favor of the human brain's pattern-finding abilities. The other problem with sleep staging based on visual inspection is low throughput. Visual inspection of long-term EEG/EMG data is time-consuming and tedious. Even an experienced human rater requires hours to score 24 h of mouse sleep data. This makes it difficult to carry out quantitative and comprehensive sleep research in animals and is hindering sleep biology from becoming a truly data-driven field.

To overcome these problems, many types of sleep staging programs have been developed since the 1960s (Drane *et al*. 1969; Larsen & Walter 1970; Smith & Karacan 1971; Martin *et al*. 1972; Kohn *et al*. 1974; Winson 1976). These methods can be divided into two processes: character extraction and sleep staging.

Character extraction identifies features from EEG/EMG to discriminate sleep stages. Along with the development of automated sleep staging programs, efforts have been made to identify the proper features for extraction. Many automated staging programs use specific bands of the EEG power spectrum as features (Johns *et al*. 1977; Chouvet *et al*. 1980; Van Luijtelaar & Coenen 1984; Stanus *et al*. 1987; Mamelak *et al*. 1988; Benington *et al*. 1994; Berthomier *et al*. 2007; Kohtoh *et al*. 2008; Brankack *et al*. 2010; Gilmour *et al*. 2010; Vural & Yildiz 2010; Pan *et al*. 2012). The most common power spectra bands in rodents are referred to as delta (0.5–4 Hz), theta (6–10 Hz) and sigma (10–15 Hz). Other characters have also been proposed, including information that is harder for humans to intuitively interpret. These include coefficients of wavelet analysis (Ebrahimi *et al*. 2008; Gabran *et al*. 2008; Sinha 2008; Fraiwan *et al*. 2010, 2011; Garces Correa & Laciar Leber 2010; Agrawal *et al*. 2011), bispectral density (Acharya *et al*. 2010; Swarnkar *et al*. 2010), parameters of multichannel autoregressive modeling (Zhovna & Shallom 2008) and matching pursuit method with slow wave patterns (Picot *et al*. 2011). Recently, two groups have proposed using a broader range of the EEG spectrum, more finely binned than the three classical bands, and reported higher-quality sleep stage classification (Vivaldi & Bassi 2006; Rytkönen *et al*. 2011). No matter what kind of staging methods are used, the number of features passed to the sleep staging step is usually important for performance. Some groups have achieved reduction of feature dimension without losing critical information using principal component analysis (Vivaldi & Bassi 2006; Gilmour *et al*. 2010) or support vector machine-based recursive feature elimination (Koley & Dey 2012).

The features abstracted from the raw dataset are then used for scoring sleep stages. Automated staging programs can be divided into two groups according to their annotation methods: unsupervised and supervised. Unsupervised staging programs use ‘hard’ rules to annotate the data. Such rules are defined before the staging process. Therefore, unsupervised staging programs do not use rules based on specific data derived from each subject. This makes the results reproducible, but sensitive to data outside the anticipated input range. However, supervised staging programs, which have recently become a popular approach, use ‘soft’ rules for annotation. They learn the proper decision rules based on a training dataset of each subject and then analyze the remaining data of the subject. This makes the staging program resilient to irregular data because the training dataset includes uncommon characters from the original data. However, a human rater should annotate the training dataset for each subject, which introduces subjectivity.

Classically, automated sleep staging programs were based on unsupervised algorithms (Larsen & Walter 1970; Smith & Karacan 1971; Martin *et al*. 1972; Kohn *et al*. 1974; Winson 1976; Johns *et al*. 1977; Mendelson *et al*. 1980; Van Luijtelaar & Coenen 1984; Kuwahara *et al*. 1988; Neckelmann *et al*. 1994; Doman *et al*. 1995; Veasey *et al*. 2000; Kohtoh *et al*. 2008). Since the late 1980s, supervised sleep staging programs incorporating sophisticated machine learning algorithms have become more popular, due to their ability to improve the accuracy of staging by including the variances between subject animals. The machine learning algorithms include neural networks (Mamelak *et al*. 1991; Robert *et al*. 1996, 1997; Schaltenbrand *et al*. 1996; Baumgart-Schmitt *et al*. 1997; Grözinger & Röschke 2002; Ebrahimi *et al*. 2008; Gabran *et al*. 2008; Hassaan & Morsy 2008; Sinha 2008; Garces Correa & Laciar Leber 2010; Fraiwan *et al*. 2011), support vector machines (Crisler *et al*. 2008; Koley & Dey 2012), training hidden Markov models (Grube *et al*. 2002; Pan *et al*. 2012) and parameter estimation of Gaussian mixture models (Acharya *et al*. 2010).

Although many of the supervised sleep staging programs have improved the throughput of sleep staging because only small amount of manual staging is required, subjectivity still remains as a problem. These methods let the machine ‘learn’ the desired sleep stage recognition rules from the dataset of EEG/EMG tagged with sleep/wake status scored by ‘human’ experts. Sleep staging by a human rater or by supervised programs can absorb many kinds of variance in the EEG/EMG data although it sacrifices the objectivity because of inter- or inner-rater differences. Therefore, an automated sleep staging program that can fully substitute manual scoring requires minimization of subjectiveness due to inter- and inner-rater differences and maximization of robustness against many sources of variability inherited in EEG/EMG data. The past automated sleep staging programs have either of four major problems in this aspect.

The first problem is related to subjectiveness. Modern automated sleep staging programs are often tied with machine learning algorithms using ‘soft’ rules, which requires supervision by a human rater. They are very robust against variance, but subjective in nature. To solve this subjectiveness problem, we adopted the classical unsupervised approach using ‘hard’ rules, which is objective in principle.

The second problem is related to robustness, one of the weaknesses in unsupervised algorithms. Most of the automated sleep staging programs, either supervised or unsupervised, attempt to stage each ‘epoch’ of EEG/EMG data, which has the minimal time length, typically 4–10 s, for staging sleep. It is not very difficult to stage an epoch that has typical waveform for a specific stage. The toughest cases are when the epoch has borderline characteristics of multiple stages because the borderline between multiple stages is very variable and therefore hard to determine. To solve this ‘borderline’ problem, we adopted the strategy introduced by Gilmour *et al*. (Gilmour *et al*. 2010). In this strategy, epochs are first clustered into a limited number of groups and then annotated based on the statistical value of each group. This ‘cluster-first’ strategy divides the borderline problem into two parts, clustering and annotation, and makes the annotation much easier and more robust because the borderline cases are already clustered into a group that has similar characters if the clustering algorithm is appropriate. Therefore, this ‘cluster-first’ strategy can convert the difficult classification problem in staging of borderline cases into the clustering problem. This conversion is beneficial because we can use recently developed sophisticated algorithms for clustering.

The third problem is also related to robustness, especially involved in clustering. Many automated sleep staging programs adopt model-based algorithms in clustering or classification. Model-based algorithms work well when the model is known *a priori*. When the system steps out from the model, characters that are extracted through the false model are useless. To solve this problem, we adopted a model-free clustering algorithm which is based on nonparametric density estimation (Azzalini & Torelli 2007).

The remaining forth problem is involved in character extraction and again related to robustness of sleep staging. Many automated sleep staging programs use limited power bands of EEG/EMG for sleep staging. They may cause problems because mouse inbred strains are known to have different distributions of EEG power bands (Franken *et al*. 1998). As mentioned above, some groups reported that the broader range of the EEG spectrum with finer bin than the three classical band contains useful information for sleep staging (Vivaldi & Bassi 2006; Rytkönen *et al*. 2011). Thus, we used comprehensive EEG/EMG power spectra with the finest bin the recorder may take. We expected that a wider range and finer bin of EEG/EMG power spectra might cover the individual variance of the subject. The use of broader and finer EEG/EMG power spectra increases the dimension of the data, which consumes the computation power and takes longer time for sleep staging. To reduce the dimension of data, we therefore performed principle component analysis of EEG/EMG power spectra in the character extraction step.

In this study, we combined solutions to all of the four problems and developed an unsupervised fully automated sleep staging program, FASTER (Fully Automated Sleep sTaging method via EEG/EMG Recordings). All efforts were made to minimize subjectivity and increase robustness against many sources of variance accompanying to the EEG/EMG data. FASTER was first developed by using the EEG/EMG data from wild-type mice and then tested for mice with drug-induced alterations in sleep/wake patterns or arrhythmic genetic modifications in a clock gene. The source code of FASTER is open source (GNU General Public License) and available for future improvement in the sleep research field.

## Results

### Basic structure of the FASTER algorithm

FASTER is composed of three major steps, character extraction, clustering and annotation (Fig. 1B). In the character extraction step (Fig. 1B, top panels), both EEG and EMG time-domain data are first split into epochs with constant time length of 8 s. This EEG/EMG time-domain data are next converted by fast Fourier transform (FFT) into frequency-domain data, the EEG/EMG power spectra. We used the power up to the maximum frequency, called Nyquist frequency (i.e., half of the sampling frequency), which corresponds to 50 Hz in this study because EEG/EMG data were recorded at 100 Hz. Characters of EEG/EMG power spectra are then extracted by principal component analysis (PCA). In the following clustering step (Fig. 1B, middle panels), all epochs are grouped together into a limited number of clusters according to the characters of EEG/EMG power spectra obtained in the character extraction step. We used the nonparametric density estimation clustering method (Azzalini & Torelli 2007) to cluster the epochs. This method uses two pieces of information for clustering. First, it estimates a probability density for each epoch by a Gaussian kernel method. Second, all epochs are connected by Delaunay triangulation, which is a method to triangulate a set of points (i.e., epochs in this study) under the criteria that the circumcircle does not include any other points. Once the information is calculated, the clusters are defined by finding subsets of high-density regions that are connected by Delaunay triangulation. Because the nonparametric density estimation clustering method itself selects the number of clusters, it is powerful to cluster certain datasets without *a priori* model or without information on the number of clusters. In this study, the model-free clustering was proper because we wanted to keep the clustering as objective as possible. In the final annotation step (Fig. 1B, bottom panels), each cluster is annotated without supervision by a human rater according to the statistical value of characteristics for each cluster. In this study, we use relative differences in the median logarithm of EMG power and EEG delta power of each cluster. Overall, all steps were fully automated and unsupervised.

### Optimization of FASTER

To optimize the parameters for hard rules in the FASTER algorithm, we first prepared an EEG/EMG dataset with manual annotation of sleep/wake stages. We recorded continuous EEG/EMG data for 6 days from four C57BL/6J mice and manually scored into three stages (NREM sleep, REM sleep and wake) according to the criteria described in the Experimental Procedures section. These annotated data were then used to determine the optimal parameter values for the FASTER algorithm by assessing its performance and computation time for different values of parameters.

Parameter values for the FASTER algorithm were optimized in three steps: character extraction, clustering and annotation. First, we optimized a parameter value for character extraction. Because the optimal degree of information extracted from EEG/EMG power spectrum is not immediately known, we optimized the number of principal components in character extraction to achieve higher performance with less computation time for sleep staging. Second, we optimized parameter values for clustering. The developers of nonparametric density estimation clustering supply a recommended shrinkage factor for the smoothing bandwidth and the optimal number of grids for the given dataset size (Azzalini & Torelli 2007). However, the clustering results are heavily dependent on the smoothing factor (bandwidth of the density estimation kernel) and the grid width of cluster core search. Therefore, the parameter values for smoothing factor and the grid width of cluster search were optimized by assessing both performance and computation time. Finally, we optimized the parameter values for annotation. As discussed above, many unsupervised staging programs use a certain threshold to classify (or annotate) each epoch into different sleep stages. However, FASTER annotates each cluster, not an epoch, by using two statistical values of each cluster: the median logarithm of EMG power and EEG delta power. We thus calculated optimal values for these parameters in annotation. In the following sections, we describe the detailed procedures to determine the optimal parameter values for FASTER.

### Optimization of character extraction: the number of principal components

**Figure 2:**
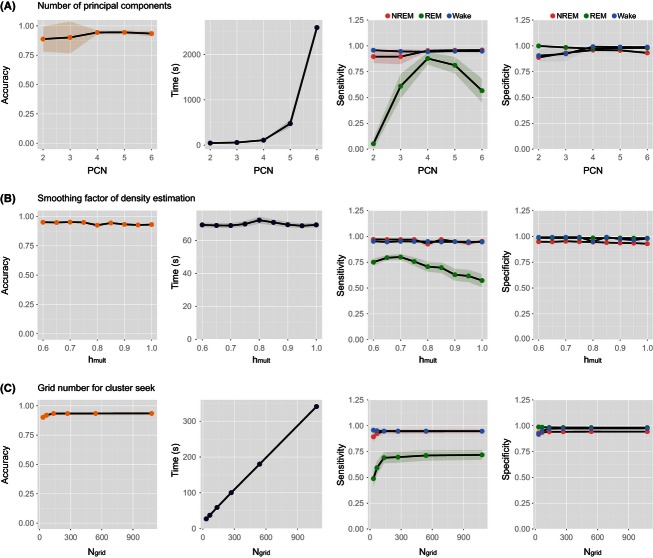
Optimization of parameter values in the character extraction and clustering steps. The panels show computation time, accuracy, sensitivity and specificity for each stage (from left). In the sensitivity and specificity panel, red, green and blue dots denote NREM sleep, REM sleep and wake, respectively. The points are mean and the shaded area denotes standard error of the mean. Every optimization is carried out using 5400 epochs randomly sampled from the 6-day-length dataset of four C57BL/6J mice. (A) Optimization of the number of principal components PCN in the character extraction step. While fixing *h*_mult_ = 0.85 and *N*_grid_ = 135 as arbitrary initial values, FASTER was tested in PCN from 2 to 6. Each PCN was evaluated four times for four different mice, resulting in 16 tests. PCN = 4 was selected for the optimal value because nearly all of the performances (e.g., accuracy, sensitivity of REM sleep) peaked at PCN = 4. (B) Optimization of the smoothing factor *h*_mult_ of probability density estimation in the clustering step. Fixing the *N*_*grid*_ = 135 and PCN = 4 as arbitrary initial values, the performance of FASTER was tested in *h*_mult_ from 0.6 to 1.0 by 0.05. Each *h*_mult_ was evaluated twelve times for four different mice, resulting in 48 tests. The computation time, accuracy and the specificity did not differ among different *h*_mult_. Because the sensitivity of REM sleep peaked at *h*_mult_ = 0.7 and the other two stages did not have change within this range, *h*_mult_ = 0.7 was selected as an optimal value. (B) Optimization of the grids numbers *N*_grid_ for cluster detection in the clustering step. While fixing *h*_mult_ = 0.85 and PCN = 4 as arbitrary initial values, FASTER was tested in *N*_grid_ from 33 to 1080. Each *N*_grid_ was evaluated twelve times for four different mice, resulting in 48 tests. Accuracy, sensitivity and specificity all reached near-plateau and keep increasing slightly when *N*_grid_ is 540 or greater. For an optimal number of grids, 540 was chosen for this data length. If there is enough computation power, picking *N*_grid_ for the same number of the dataset will be the best choice because theoretically it should provide the best results.

The parameter optimized in the character extraction step is the number of principle components passed to the subsequent steps. In this study, the top four principal components are sufficient to express over 38.4% of the original EEG/EMG power spectrum's variance (Fig. S1 in Supporting Information). However, the optimal number of components to adopt in the subsequent steps is not immediately obvious. Therefore, we compared the performance of staging results and computation time with different principal component numbers (PCN). When PCN is in the range of 2–6, the accuracy, sensitivity of NREM sleep and wake and specificity of all three stages reach to near-plateau, especially when PCN is greater than 4. Meanwhile, computation time exponentially increases along the increase in PCN. However, the sensitivity of REM sleep showed a peak at PCN = 4. According to these results (Fig. 2A), PCN = 4 was chosen as an optimal number of principal components.

### Optimization of clustering: the smoothing factor of density estimation

The clustering algorithm first estimates the probability density from the EEG/EMG dataset. Therefore, we first optimized the smoothing factor, which is a critical parameter in probability density estimation. A Gaussian kernel method is used for the nonparametric density estimation in the clustering step. Estimating the probability density of a dataset is like rescaling and smoothing the dataset. When the dataset *x* has *N* points of *d* dimensional data and the Gaussian kernel which has a bandwidth *h*, the estimated probability density 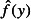 is given by:


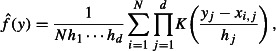



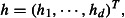



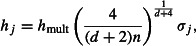



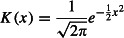


Azzalini and Torelli observed that shrinking *h* slightly toward zero is often advantageous and recommend the shrinkage factor *h*_mult_ of 0.75 (Azzalini & Torelli 2007). Because the number of cluster that can be detected is heavily dependent on smoothness of the estimated probability density, we optimized *h*_mult_ to improve the accuracy of the staging results (Fig. 2B). The smaller the bandwidth is, the shaggier the estimated probability density would be. Especially when *h*_mult_ is smaller than 0.4, the number of the clusters, which are the number of peaks in the estimated probability density, did not match with the number of the detected saddle points (usually, the number of peaks and saddles matches). Therefore, the clustering program was not able to complete (Fig. S2 in Supporting Information). If the bandwidth is too large, the cluster number will decrease and detected clusters might not be sufficient to adequately describe the dataset. We have chosen the optimal *h*_mult_ as 0.70 because the accuracy was constantly high through the range of 0.60 < *h*_mult_ < 0.85 and the sensitivity of REM sleep was highest at *h*_mult_ = 0.70, whereas sensitivity of NREM sleep and wake did not change much in this range (Fig. 2B).

### Optimization of clustering: the grid numbers for cluster detection

After the calculation of estimated probability density as described above, the clustering algorithm then searches for cluster cores in the estimated probability density. Therefore, the second optimized parameter in the clustering step is the number of grids *N*_grid_, which is a critical parameter in the detection of cluster cores. Supposed the dataset has *N* points in total, the clustering algorithm scans every *N/N*_grid_ points in the dataset from the point of maximum probability density (Fig. S3 in Supporting Information). Theoretically, scanning every point in the dataset (i.e., *N*_grid_ *= N*) will maximize the capability in detecting cluster cores although it also costs the maximum computation power. Practically, sufficiently large number of *N*_grid_ grids will give acceptable results. Therefore, we optimized *N*_grid_ as smaller as possible by maximizing performance of sleep staging along with minimizing the computation time (Fig. 2C). The accuracy gets nearly constant when *N*_grid_ is larger than 135, as well as the sensitivities of NREM sleep and wake. Meanwhile, REM sleep does not reach to plateau unless *N*_grid_ is greater than 540. Considering its performance and computation time, we chose 540 as the optimal *N*_grid_. Because the total points in the dataset were 5400 in this optimization, this grid number denotes that the probability density is scanned through every 10 points. This is finer than the original clustering method, which uses default *N*_grid_ of 50 for any size of the data which has more than 50 data points (Azzalini *et al*. 2012).

### Optimization of annotation

**Figure 3:**
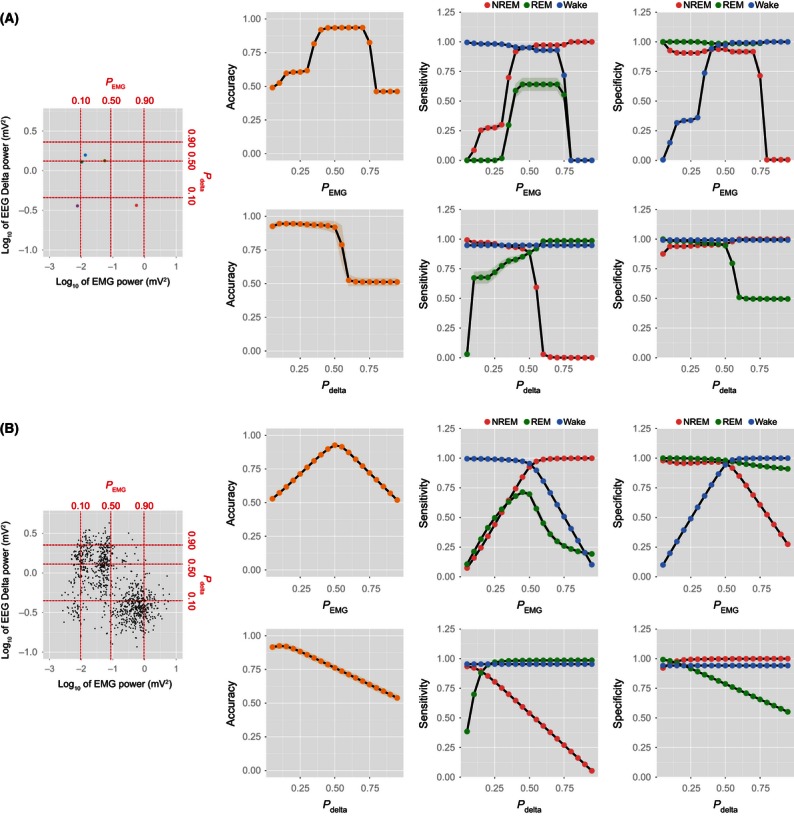
Optimization of parameter values in the annotation step. In the sensitivity and specificity panel, red, green and blue dots denote NREM sleep, REM sleep and wake, respectively. The points and the shaded area in the three right columns denote mean and standard error of the mean, respectively. Every optimization is carried out using 5400 epochs randomly sampled from the 6-day-length dataset of four C57BL/6J mice. (A) The left panel shows a schematic view of the relationship between median logarithm of EMG power and EEG delta power and quartile of logarithm of EMG power (*P*_EMG_) and nonwake epochs’ EEG delta power (*P*_delta_). Each dot represents different clusters. In this case, there are five clusters. The upper row of the right panels shows results of the annotation when *P*_EMG_ is tested from 0.05 to 0.95 by 0.05 and the *P*_delta_ is fixed to 0.1. The lower row of the right panels shows results of annotation when *P*_delta_ is tested from 0.05 to 0.95 by 0.05 and the *P*_EMG_ is fixed to 0.5. Each *P*_EMG_ and *P*_delta_ was evaluated twelve times for four different mice, resulting in 48 tests. The accuracy was maximized when *P*_delta_ is from 0.45 to 0.70 and *P*_delta_ is from 0.10 to 0.45, respectively. (B) Each dot in the left panel represents different epochs before clustering. The upper row of the right panels shows results of annotation when *P*_EMG_ is tested from 0.05 to 0.95 by 0.05 and the *P*_delta_ is fixed to 0.1. The lower row of the right panels shows results of annotation when *P*_delta_ is tested from 0.05 to 0.95 by 0.05 and the *P*_EMG_ is fixed to 0.5. Each *P*_EMG_ and *P*_delta_ had evaluated 12 times for four different mice, resulting in 48 tests. The accuracy was maximized when *P*_EMG_ is 0.5 and *P*_delta_ is 0.10, respectively. We chose this number because this value gave optimal results in the annotation of the clustered dataset (see (A)).

The annotation step in FASTER involves assigning the proper sleep/wake stages to the limited number of clusters. Sleep stages can be characterized by relative difference in EMG power and EEG delta power. If each cluster is mainly made of the same stage, the median logarithm of EMG power and EEG delta power are sufficient to discriminate sleep/wake stages. Therefore, we adopted the method to annotate the clusters by their statistical values (i.e., median logarithm of EMG power and EEG delta power). The advantage of this approach is that we do not need to care about the borderline epochs; at least the cluster is guaranteed to have mostly epochs with the identical stage. If the median logarithm of EMG power of a cluster is larger than the *P*_EMG_ quartile of the total EMG power, the cluster is annotated as ‘wake’. The remaining clusters are annotated as ‘NREM sleep’ if the median logarithm of EEG delta power of the cluster is greater than *P*_delta_ quartile of the nonwake epochs and as ‘REM sleep’ when it is not. We compared the accuracy of staging results for different *P*_EMG_ and *P*_delta_ (Fig. 3A). For annotating ‘wake’ and ‘nonwake’ status, the accuracy was maximized when 0.45 < *P*_EMG_ < 0.70. For annotating ‘NREM sleep’ and ‘REM sleep’, the accuracy was maximized when 0.05 < *P*_delta_ < 0.45. In this study, we chose 0.5 for *P*_EMG_ and 0.1 for *P*_delta_ because these values give the maximum accuracy when we used these values for nonclustered epochs (Fig. 3B).

**Figure 4:**
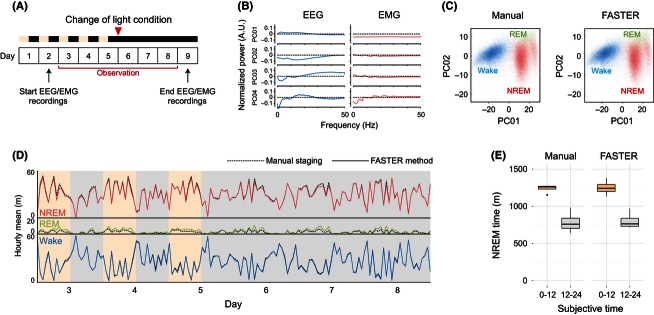
Results of staging C57BL/6J mice with FASTER. (B), (C) and (D) show a representative mouse. Results for the other three animals are found in Supporting Information (Fig. S3A). In (A) and (D), orange and black boxes denote light on and off, respectively. (A) Experimental scheme. (B) Eigenvectors of the top four principal components. (C) Scatter plot of the first two principal components with staging results. The left and right panels show the results from manual staging and FASTER, respectively. (D) Time spent in each stage per hour during experiments. The dashed and solid lines show the manual staging and the staging by FASTER, respectively. (E) The total NREM time during subjective day and night during 3 days of constant darkness. Both in manual and in FASTER staging, mean NREM sleep time in subjective day and night was significantly different (*P* = 0.0012 and *P* = 0.0012 for manual staging and FASTER by the unpaired Student's *t*-test).

Overall, we optimized FASTER for the number of principal components to pass to subsequent procedures, the smoothing factor for probability density estimation, the number of grids to seek the cluster peaks and the thresholds for annotation. Because these optimizations were carried out in parallel, every optimized parameter was combined to evaluate the maximal functionality of the FASTER algorithm, and the analysis was redone in C57BL/6J male mice that were used for optimization. These animals were recorded in a 12-h light/dark condition for 3 days, followed by a constant darkness for 3 days (Fig. 4A). The first PC has larger vector elements in the EMG than in the EEG (upper row of Fig. 4B and Fig. S4A in Supporting Information). The second, third and forth PCs have relatively smaller elements in EMG and unique distribution of elements in EEG (the lower three rows of Fig. 4B and Fig. S4A in Supporting Information). Using these principal components, FASTER divided the epochs into three sleep/wake stages (Fig. 4C, D and Fig. S4A in Supporting Information) and the accuracy was 94.6 ± 1.4%, the sensitivity and specificity for each stage were 96.2 ± 1.7% and 95.6 ± 3.1%, 87.1 ± 3.3% and 97.8 ± 1.1%, 94.2 ± 3.3% and 99.2 ± 0.3% for each NREM, REM and wake status, respectively (Table 1 and Table S1 in Supporting Information). The average time spent to analyze a 24 h of mouse data was 605.7 ± 13.9 s using a laptop computer with a single processor. One of the features in wild-type animals is that they exhibit their circadian sleep/wake rhythm, even in constant darkness. They have a robust internal time, which is called circadian time in this study. Figure 4E shows that FASTER was able to detect the NREM sleep time difference in the subjective day and night under constant darkness condition. The high accuracy suggests combining in-parallel optimization worked well in FASTER.

**Table 1:** Summary of the FASTER performance tests. Basal: spontaneous sleep/wake cycle under 12:12-h light/dark cycle for the first 3 days followed by constant darkness for 3 days; MOD-IP: modafinil IP experiment to induce excessive wakefulness; DIP-IP: diphenhydramine IP experiment to induce excessive sleepiness. Each value represents the mean ± SD

			NREM		REM		Wake		
						
Strain	Experiment	Number of mice	Sensitivity	Specificity	Sensitivity	Specificity	Sensitivity	Specificity	Accuracy
C57BL/6J	Basal	4	96.2 ± 1.7%	95.6 ± 3.1%	87.1 ± 3.3%	97.8 ± 1.1%	94.2 ± 3.3%	99.2 ± 0.3%	94.6 ± 1.4%
C57BL/6J	MOD-IP	3	96.6 ± 1.5%	90.9 ± 4.0%	60.5 ± 13.3%	98.7 ± 0.2%	92.2 ± 2.2%	98.1 ± 0.8%	91.9 ± 2.5%
C57BL/6J	DIP-IP	3	96.1 ± 1.8%	93.7 ± 0.6%	71.7 ± 6.1%	98.2 ± 0.2%	92.8 ± 0.8%	98.4 ± 1.5%	93.2 ± 1.0%
*Bmal1*^*−/−*^	Basal	3	95.1 ± 4.3%	92.4 ± 2.6%	63.4 ± 17.6%	98.4 ± 2.2%	94.3 ± 0.8%	98.5 ± 0.7%	92.6 ± 1.1%

### Staging drug-induced overwaking and oversleeping mice with FASTER

**Figure 5:**
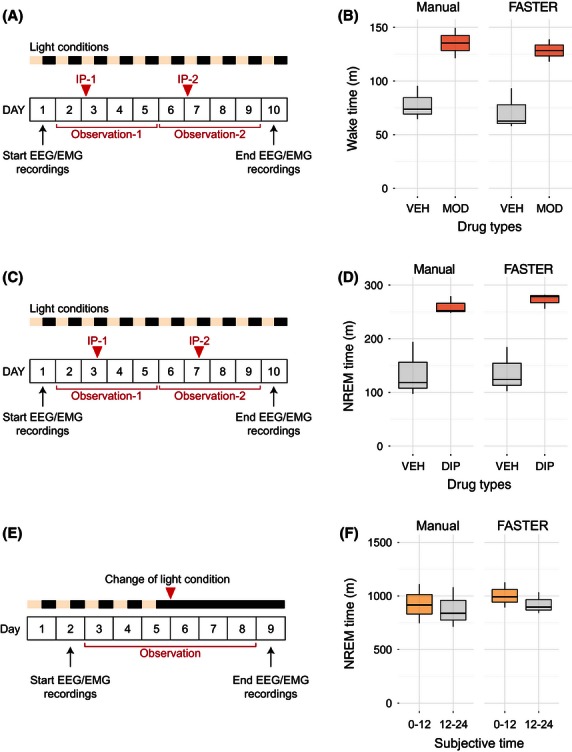
Results of performance test of FASTER in various conditions. (A) Experimental scheme of staging modafinil induced prolonged wakefulness in C57BL/6J mice with FASTER. (B) The total wake time for three hours after the IP. Both in manual and in FASTER staging, it was possible to detect significantly longer mean wake time in the modafinil-administered group (*P* = 0.016 and *P* = 0.018 for manual staging and FASTER by the unpaired Student's *t*-test.). (C) Experimental scheme of staging diphenhydramine-induced prolonged sleepiness in C57BL/6J mice with FASTER. (D) The total NREM time for six hours after the IP. Both in manual and in FASTER staging, it was possible to detect longer NREM time in the diphenhydramine-administered group (*P* = 0.016 and *P* = 0.0064 for manual staging and FASTER by the unpaired Student's *t*-test.). (E) Experimental scheme of staging genetically modified circadian mutant *Bmal1*^*−/−*^ mice with FASTER. (F) The total NREM time during subjective day and night during 3 days under constant darkness. Both in manual and in FASTER staging, it was unable to detect significant difference in NREM time between subjective day and night (*P* = 0.77 and *P* = 0.42 for manual staging and FASTER by the unpaired Student's *t*-test.).

Because our final goal is to evaluate animals with unnatural sleep/wake status by FASTER, we tested our method on animals with drug-induced sleep/wake status. In other words, this examines the robustness of FASTER when the sleep/wake ratio has changed temporally in a certain period of the experiment. First, we examined prolonged wakefulness by injecting modafinil into the intraperitoneal cavity to induce wakefulness through dopaminergic transporter inhibition (Qu *et al*. 2008). During EEG/EMG recording, we injected modafinil intraperitoneally to mice at the beginning of the light phase (Fig. 5A) when mice have tendency to sleep. After manually staging the data (8-day-length, *n* = 3), we compared the staging results derived from FASTER (Fig. S4B in Supporting Information). The increase in wake induced by modafinil injection was detected both in manual staging and in FASTER (Fig. 5B). The total accuracy was 91.9 ± 2.5% and the sensitivity and specificity for each stage were 96.6 ± 1.5% and 90.9 ± 4.0%, 60.5 ± 13.3% and 98.7 ± 0.2%, 92.2 ± 2.2% and 98.1 ± 0.8% for each NREM, REM and wake status, respectively (mean ± SD, 8-day-length, *n* = 3, Table 1 and Table S1 in Supporting Information). Next, we examined prolonged sleepiness by using diphenhydramine, which is a histamine H1 receptor antagonist. Diphenhydramine causes sleep by blocking the wakefulness maintenance pathway of histamine (Saitou *et al*. 1999). Under EEG/EMG recording, we injected diphenhydramine intraperitoneally to mice at the beginning of the dark phase, which is the active phase for nocturnal animals (Fig. 5C). After manual staging (8-day-length, *n* = 3), the results were compared with FASTER-derived stages (Fig. S4C in Supporting Information). The increase in NREM sleep time after diphenhydramine injection was clearly detected by both manual staging and FASTER (Fig. 5D). The accuracy for these mice was 93.2 ± 1.0% and the sensitivity and specificity for each stage were 96.1 ± 1.8% and 93.7 ± 0.6%, 71.7 ± 6.1% and 98.2 ± 0.2%, 92.8 ± 0.8% and 98.4 ± 1.5% for each NREM, REM and wake status, respectively (mean ± SD, 8-day-length, *n* = 3, Table 1 and Table S1 in Supporting Information). These experiments show that FASTER is capable of detecting not only normally distributed sleep/wake status but also temporarily altered sleep/wake status.

The EEG/EMG recording data of six C57BL/6J mice obtained in these overwaking or oversleeping drug administration experiments can be also used for the validation of FASTER in wild-type mice at normal condition. One-day-length of EEG/EMG recordings during the previous day of drug administration that was recorded for control were evaluated for this purpose. Because each mouse had two chances of administration (vehicle vs. overwaking drug, or vehicle vs. oversleeping drug), total length of 2 days were used for the test per animal. In these mice, the total accuracy was 92.5 ± 1.3% and the sensitivity and specificity for each stage were 95.4 ± 1.2% and 93.6 ± 2.6%, 66.5 ± 10.1% and 98.5 ± 0.5%, 93.2 ± 1.2% and 98.6 ± 1.0% for each NREM, REM and wake status, respectively (mean ± SD, 2-day-length, *n* = 6). Taken together, these results confirm that FASTER is able to detect sleep/wake stages accurately in wild-type animals, in either normal or drug-administrated conditions, without human assistance. These results are much objective and the time spent for staging is faster in comparison with manual staging by human raters.

### Staging genetically modified animals with FASTER

One of the motivations behind FASTER is to increase the throughput of sleep staging, for example, to screen a large number of genetically modified mice for defects in sleep. As a proof of principle, we examined a genetically modified strain with severe circadian abnormalities – mice that lack circadian rhythm in their sleep/wake behavior. Testing FASTER in these arrhythmic mice will evaluate the robustness of this method against genetically modified mice with sleep/wake phenotypes. We used *Bmal1*^*−/−*^ mice, which are known to exhibit an arrhythmic sleep/wake pattern (Laposky *et al*. 2005). EEG/EMG from three male *Bmal1*^*−/−*^ mice was recorded while they were kept in 12-h light/dark condition for the first 3 days followed by another 3 days under constant darkness (Fig. 5E). Manual staging and results obtained by FASTER were compared (Fig. S4D in Supporting Information). FASTER showed no significant difference in NREM sleep time between subjective day and night in *Bmal1*^*−/−*^ mice during the constant darkness condition (Fig. 5F), which is one of the major characteristics found in mice lacking circadian clock. This arrhythmic phenotype is markedly different from the circadian sleep/wake pattern in wild-type mice under the constant darkness condition (Fig. 4E). The total accuracy in *Bmal1*^*−/−*^ mice was 92.6 ± 1.1%. Taken together, our results show that FASTER can reliably detect unnatural sleep/wake durations and distributions in both drug- and genetically perturbed animals, which are key factors for screening mice with sleep/wake abnormalities.

## Discussion

We have developed FASTER, an unsupervised fully automated sleep staging method for mice. This method reports sleep/wake stages by analyzing the comprehensive power spectrum of EEG and EMG. Although there are other semi-automated staging programs, no system has achieved a fully automated sleep staging functionality. FASTER is not only fully automated, but the accuracy is comparable to the semi-automated staging programs and it is much faster than manual staging by visual inspection.

The basic strategy of FASTER is to use the classical ‘hard’ rule, which is objective but prone to variance within subjects. To adopt the classical hard rule, we have deliberately divided the ‘classification’ into ‘clustering’ and ‘annotation’. The FASTER algorithm thereby absorbs the individual variance as much as possible in the character extraction and clustering steps to safely adopt the classical ‘hard’ rule in annotation step. For example, in the character extraction step, FASTER uses comprehensive EEG/EMG power instead of band-specific powers (e.g., EEG delta power), which is often used in many manual and automated stagers. The use of comprehensive EEG/EMG power could cover and hence absorb the individual variance among subjects. This ‘full power spectrum’ strategy is based on the report on the interstrain variance in distribution of classical power bands (Franken *et al*. 1998), as well as other reports on that expanded bands beyond the classical ones are informative for staging accuracy (Vivaldi & Bassi 2006; Rytkönen *et al*. 2011). In this aspect, two improvements might be possible in the future to further cover and absorb the variance among subjects. First, using faster sampling to gather higher powers as characters might detect intersubject variance. In the current study, we have used 100-Hz sampling for EEG/EMG recordings that limits the available power bands of both signals up to the Nyquist frequency, 50 Hz. Because some eigenvectors of the principal components include large elements in the high end of the power spectrum (e.g., see the third eigenvector in Fig. 4B), higher-frequency powers might have useful information for sleep staging. Second, information on the transition of states might absorb variance among subjects. In FASTER, the order of the epochs does not affect the staging results. When human raters stage the time series of EEG/EMG, it is natural to take information on the previous epochs (e.g., REM sleep tends to occur after NREM sleep, but not after wake stage). Therefore, it will be an important future work to take the order information into account in the character extraction step.

In the clustering step, we have adopted the clustering algorithm based on nonparametric estimation of probability density (Azzalini & Torelli 2007) to avoid subjectiveness from holding up a model before clustering. This clustering method makes no assumptions about the distribution of data and chooses the number of clusters automatically. One of the few disadvantages of this algorithm is that it is fairly computationally expensive. Related to this higher computational cost, we need to point out two limitations in this study, both of which can be solved in near future by the improvement of computers. First, because we aimed a handy system, we implemented FASTER in a free software environment R (R Core Team 2012) and ran on a laptop computer with a single processor. This resulted in the maximum clustering size at once to be 5400 epochs practically. For example, if the total data length is 86 400 epochs (8-day-length data when one epoch is 8 s), the data are divided into 16 groups and clustered independently. In this way, 24 h of EEG/EMG recorded from single mouse can be staged in 10 min. However, if 8-day-length data are clustered directly, approximately 6 h is expected for staging 1-day-length of data even ignoring other hardware concerns (e.g., memory size). Ideally, all data should be processed at once which will be practical in the future with the increase in speed of computers. Second, the number of grid for scanning clusters was optimized maximizing the performance while minimizing the computation time. Theoretically, the capability in detection of cluster cores is maximized when all of the data points are evaluated through the cluster scan. Therefore, the recommended number of grids of this clustering might be the number of the data points in the future.

In the annotation step, we adopted a classical ‘hard’ rule-based selection, which is objective but sensitive to variance among subjects. This employment was possible in FASTER algorithm as the results of the absorbance of the individual variance in the character extraction and clustering steps as discussed above. This allowed us to use very simple ‘hard’ rules to annotate clusters with satisfactory performance.

The robustness of FASTER was tested in drug-induced unusual sleep/wake conditions and genetically perturbed animals. The results of satisfactory detection of drug-induced prolonged wakefulness and sleepiness show the ability to stage temporarily sleep/wake differences. Because we have used circadian mutant *Bmal1*^*−/−*^ mice, which have arrhythmic sleep/wake activity, the high performance of FASTER against these genetically modified animals not only shows potential to evaluate mutants but also implies the ability to detect unnatural sleep/wake distributions. In each experiment, FASTER was comparable to manual staging, which suggests the robustness of the method and the potential of this method to analyze animals with unknown sleep/wake status.

In comparison with the latest supervised sleep programs for rodents, FASTER has comparable accuracy but lower sensitivity in REM sleep detection. For example, using approximately 5% of human rater scored data as a training dataset, Rytkönen *et al*. (2011) achieved accuracy of 93% in rats and in mice, which is comparable to our results. Their method, however, was able to detect REM sleep in sensitivity of 89%, which is higher than our optimized results (Table 1 and Table S1 in Supporting Information). In this study, we used full EEG/EMG power spectra in the character extraction step and then maximized not only total accuracy but also sensitivity of REM sleep by optimizing possible critical parameters in this scheme. Therefore, we expected that, to further increase the sensitivity of REM sleep, it might be important to use additional characters, which can efficiently distinguish REM sleep from other stages. As mentioned above, the candidates for additional characters are higher-frequency power spectrum or order information of stages. The improvement of REM sleep sensitivity is at the top of the list for future works.

We believe at least two points need to be tested in the future to further confirm the robustness of FASTER. First, we used diphenhydramine to induce prolonged sleep. However, this drug is not a major choice for insomnia patients. To confirm the robustness of FASTER, other drugs for insomnia, that is, benzodiazepines, should be tested in the future. Second, we used *Bmal1*^*−/−*^ mice as a sleep disorder model. In this strain, the sleep/wake distribution is very different from wild-type mice because of the clear disorientation in their circadian rhythm. Therefore, other models characterized by disorganized or fragmented sleep architecture, which has intact circadian rhythms, such as narcoleptic mice, should be tested in the future to challenge the robustness of FASTER in genetically modified mice.

In this study, we have developed FASTER, which is an unsupervised fully automated sleep staging method for mice based on comprehensive EEG/EMG recordings. Full automation was achieved combining the classical ‘hard’ rule-based annotation with the modern comprehensive character extraction along with model-free clustering. FASTER has comparable accuracy to conventional visual inspection-based manual sleep staging method, and it is quicker than manual inspection. All of the source codes of FASTER are fully available because we believe this automated staging program has potential to free many sleep researchers from manual sleep staging labor and making the source code freely available is a simple, but effective way to dedicate to the field of animal sleep research. Therefore, FASTER has the potential to enable data-driven quantitative and comprehensive sleep research.

## Experimental procedures

### Animal preparation and data collection

Four C57BL/6J mice (14 weeks old at recording) were used for optimization of FASTER. To test the method, a total of 9 mice were used to represent different animal groups. These mice include two groups of C57BL/6J mice that were administrated drugs (modafinil, diphenhydramine, 3 mice each, 11–13.4 weeks old at recording) by intraperitoneal injection and three *Bmal1*^*−/−*^ mice (Shimba *et al*. 2011) (12–14 weeks old at recording), which are circadian mutants. All C57BL/6J mice were purchased from Oriental Yeast Co., Ltd. (Itabashi-ku, Tokyo, Japan). See Table 1 for a summary of experiments. Procedures involving animals and their care were performed according to the RIKEN Regulations for Animal Experiments (approval ID: AH18-01-19). We anesthetized animals and implanted telemetry transmitters (Model F20-EET, DSI, St. Paul, MN, USA) for simultaneous recording of two biopotentials (EEG and EMG). Two stainless-steel screws (1 mm diameter) were soldered to the wires of telemetry transmitters and inserted through the skull of the cortex (anteroposterior, +1.0 mm; right, +1.5 mm from bregma or lambda) and served as EEG electrodes. Two other wires from the transmitter were placed into trapezius muscles serving as EMG electrodes. Animals were allowed at least 10 days to recover from surgery. EEG/EMG data were recorded wirelessly with food and water available *ad libitum*. The EEG/EMG data collecting system consisted of transmitters, an analog-digital converter and a recording computer. Sampling rate was 100 Hz for both EEG and EMG. Gold Acquisition (version 4.00, DSI) was used for the recording. The EEG/EMG data were converted to ASCII format, and both the manual and the automated sleep staging were carried out in the originally developed software on Ruby on Rails, ver. 3.1 (Rails Core Team 2011) and R (R Core Team 2012).

### Optimization of FASTER

The animals were housed in an insulated soundproof recording chamber maintained at an ambient temperature of 21 °C with a relative humidity of 50%. The chamber was light controlled under 12-h light/12-h dark cycle (light on at 6:00 A.M.) for the first 5 days followed by 4 days of constant darkness. The recording was started on the second day. The data analysis was carried out by the data recorded 6 days from the 6 A.M. of the third day (Fig. 4A).

### Drug administration

Mice were housed in the same environment as in the recordings for FASTER optimization for 10 days, and EEG/EMG was recorded for 8 days from 6:00 A.M. of the second day. The light was controlled under 12-h light/12-h dark cycle (light on at 6:00 A.M.) through the experiment. In the prolonged wakefulness experiment (Fig. 5A), modafinil (M6940-50MG, Lot#029K4618, Sigma-Aldrich, St Louis, MO, USA) was dissolved to be 1% in sterile natural saline containing 10% DMSO (06593-54, Lot#LZR7072, NACALAI TESQUE, INC., Nakagyo-ku, Kyoto, Japan) and 2% cremophor EL (C5135-500G, 1439553-13509161, Sigma-Aldrich) immediately before use and administered intraperitoneally at 8:00 A.M. (2:00 in circadian time) on the experimental day at dose of 0.3 mL per mouse. The control group was administered vehicle at a dose of 0.3 mL per mouse. Every mouse had two opportunities for intraperitoneal injection during the experiment (2:00 in circadian time on the third or seventh day), and if the animal was injected modafinil on the first chance, vehicle was injected on the second and vice versa. In the prolonged sleepiness experiment (Fig. 5C), diphenhydramine (D3630-5G, Lot#040M0205V, Sigma-Aldrich) was dissolved to be 0.2% to sterile water immediately before use and administered intraperitoneally at 7:00 P.M. (13:00 in circadian time) on the experimental day at a dose of 0.3 mL per mouse. The control group was administered vehicle at a dose of 0.3 mL per mice. As in the modafinil injection, all mice had two opportunities for injection (13:00 in circadian time on the third or seventh day), and if the animal was injected diphenhydramine on the first chance, vehicle was injected on the second and vice versa.

### Genetically modified animals

Mice were housed in the same environment as in the recordings for FASTER optimization for nine days. The light control of the chamber was identical to the FASTER optimization recordings as well (Fig. 5E).

### Data analysis

We have used R (R Core Team 2012) on MacBook Air (1.8 GHz Intel Core i7, 4 GB 1333 MHz DDR3, Mac OS X Lion 10.7.5) for every analysis. For both manual and automated staging, data were scored in 8-s epochs. In manual scoring, each epoch was staged by visual inspection as NREM, REM and wake by the following criteria: NREM sleep was characterized by high-amplitude EEG delta waves (0.5–4 Hz) with low EMG power, REM sleep was characterized by low-amplitude, high-frequency EEG theta waves (6–10 Hz) with very low EMG power, and wake was characterized by high and varying EMG power. In the automated staging, EEG/EMG data were divided into 8-s epochs and the power spectrum was computed after detrended by subtracting estimated simple linear regressions followed by the production of a Hann window. Both EEG and EMG powers from the same epoch were connected in tandem, resulting in combined EEG/EMG data. Because the sampling rate was 100 Hz, these 8 s of EEG/EMG combined power spectra had 798 columns after removal of direct current amplitudes (the first column in FFT results). If the logarithm to base 10 of the sum of EEG or EMG power is either above 1 or 2, the epoch was annotated as ‘dirty’ data and ignored for automated staging. The logarithm to base 10 of every power for every epoch was computed. By subtracting the mean and dividing by the standard deviation of these values, every power was normalized within the epoch. Then, the principal component analysis was performed on the normalized EEG/EMG combined power dataset to reduce the dimension. The top four principal components were used for clustering giving two parameters, the smoothing factor and the grid numbers, to the *pdfCluster* library (Azzalini & Torelli 2007). The number of principal components handed to the clustering library was set to 4 based on optimization. Furthermore, the parameters given to the clustering library were optimized by simulation study. The smoothing factor *h*_mult_ was set to 0.7 and the number of grids *N*_grid_ was set to 540 when the dataset size is 5400. Because the clustering time increases in an exponential manner against the data points, we decided to resample the data and divide the dataset into subsets which has less size than 5400 points. When the original data are *X* = {*x*_*1*_*,…, x*_*M*_} and dividing *X* into *k* subsets, the subset *X*_*i*_ that has *N* data points (*N* = 5400 in this study) will be defined as *X*_*i*_ = {*x*_*i*_*, x*_*i+k*_*,…*, *x*_*i+(N-1)k*_} where *k* = *M/N* and *i* = {1,*…*, *k*}. The clusters were annotated by the following rules which were also optimized based on simulation. First, the median logarithm of EMG power of each clusters was computed and clusters that have higher median logarithm of EMG power than the optimized threshold 0.5 quartile of that of all data points were annotated as ‘wake’. Within the remaining clusters, the median logarithm of EEG delta power (0.5–4 Hz) was calculated, and if it was greater than the optimized threshold 0.1 quartile of EEG delta power among all nonwake data points, it was annotated as ‘NREM sleep’ and the rest of the clusters were annotated as ‘REM sleep’.

### Performance tests

The accuracy is defined by the ratio of epochs that have agreement with manual and automated staging within the total number of epochs. The sensitivity of stage *X* is defined by the ratio of correctly staged *X* by automated staging within the total number of *X* of manual staging. The specificity of stage *X* is defined by the ratio of correctly staged *non-X* of automated staging within the total number of *non-X* of manual staging.

## Supporting Information

**Figure S1** Ratio of eigenvalues of each principal component within the variance of the original signal.

**Figure S2** Optimization results when smoothing factor of density estimation *h*_mult_ is selected from 0.2 to 1.4 by 0.2.

**Figure S3** Schematic view of nonparametric density estimation clustering (Azzalini & Torelli 2007).

**Figure S4** Individual results of FASTER, which were not shown in main figures.

**Table S1** Results from all recordings in this study

**Data S1** Source code of FASTER written in R (R Core Team 2012).
