# Supplementary material for: FASTER: an unsupervised fully automated sleep staging method for mice
Source: Genes Cells. 2013 Apr 28;18(6):502–18. doi: 10.1111/gtc.12053 (PMC3712478; doi:10.1111/gtc.12053)
Supplement: Supplementary file 1 [file gtc0018-0502-SD1.pdf]

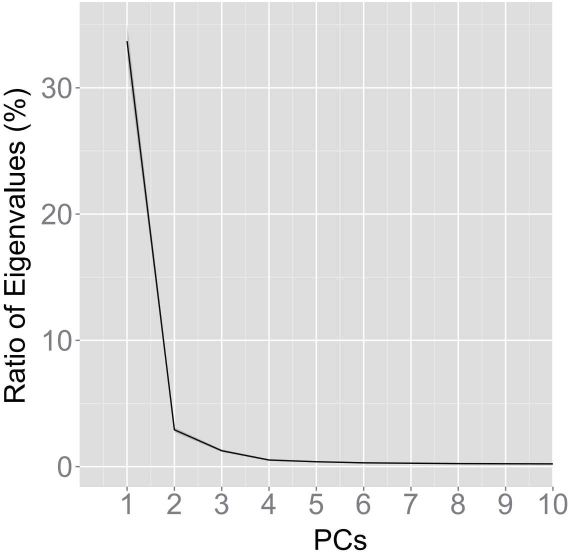

Figure S1 Ratio of eigenvalues of each principal component within the variance of the original signal. The top four components are sufficient to express over 38.4% of the original signal's variance.
