# Supplementary material for: FASTER: an unsupervised fully automated sleep staging method for mice
Source: Genes Cells. 2013 Apr 28;18(6):502–18. doi: 10.1111/gtc.12053 (PMC3712478; doi:10.1111/gtc.12053)
Supplement: Supplementary file 2 [file gtc0018-0502-SD2.pdf]

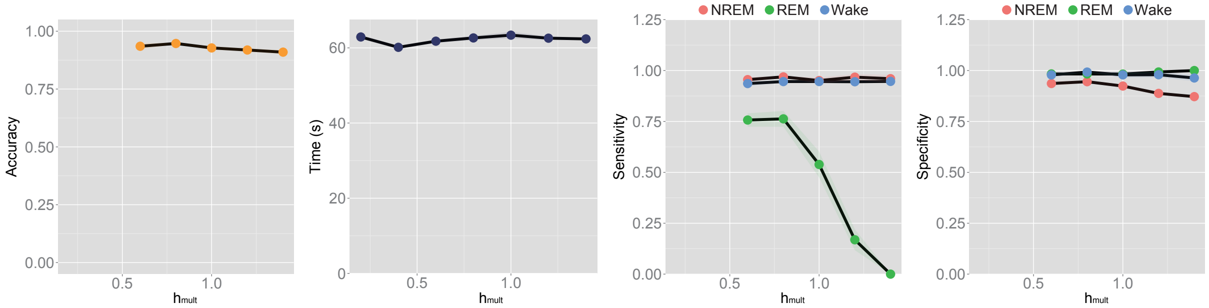


**Figure S2** Optimization results when smoothing factor of density estimation $h_{mult}$ is selected from 0.2 to 1.4 by 0.2. The panel shows computation time, accuracy, sensitivity and specificity for each stage (from left). In the sensitivity and specificity panel, red, green and blue dots denote NREM sleep, REM sleep and wake, respectively. The points and the shaded area denote mean and standard error of the mean, respectively. Every optimization is done using 5400 epochs randomly from the 6-days-length dataset of four C57BL/6J mice.
