# Supplementary material for: FASTER: an unsupervised fully automated sleep staging method for mice
Source: Genes Cells. 2013 Apr 28;18(6):502–18. doi: 10.1111/gtc.12053 (PMC3712478; doi:10.1111/gtc.12053)
Supplement: Supplementary file 3 [file gtc0018-0502-SD3.pdf]

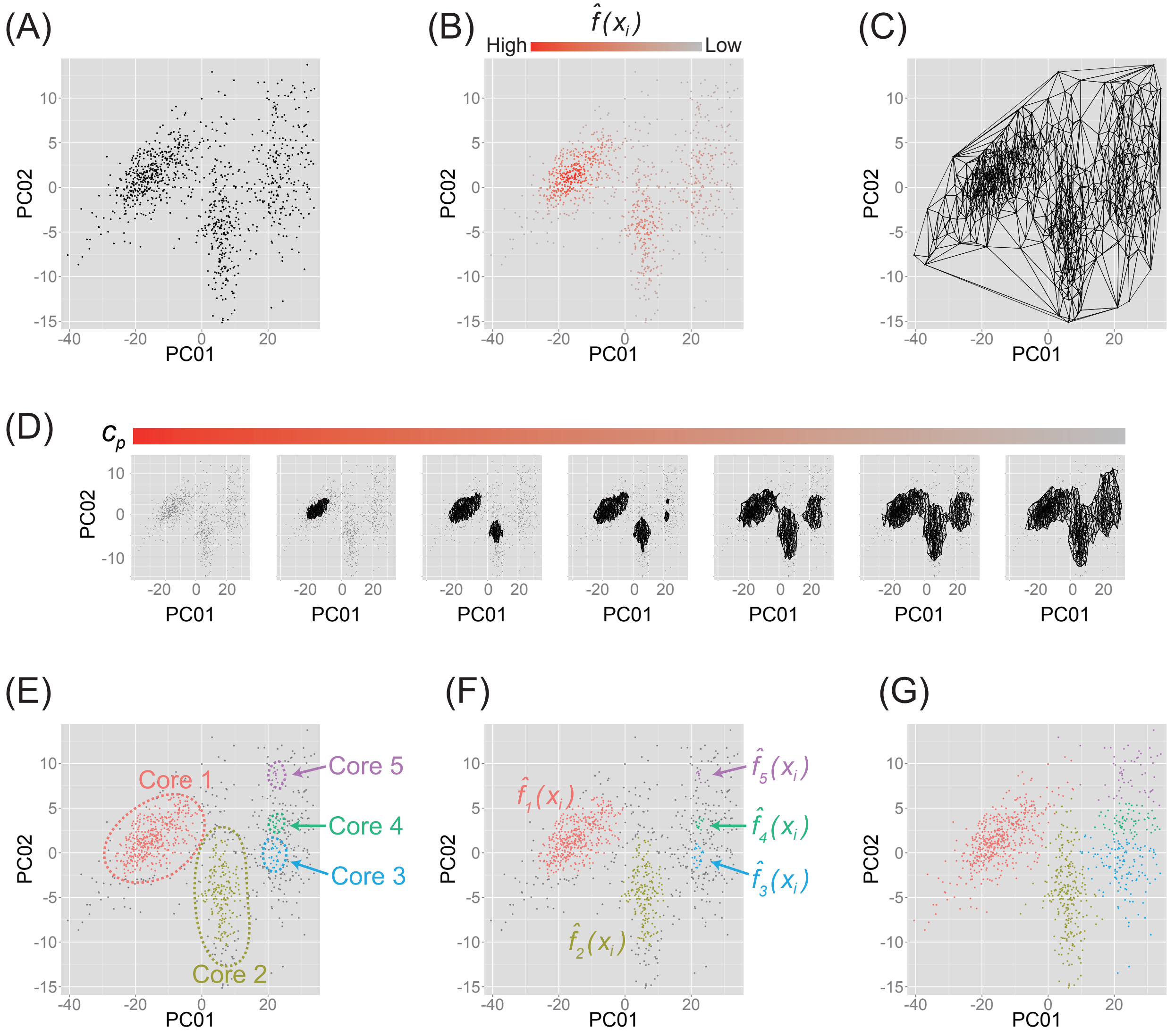

Figure S3 Schematic view of nonparametric density estimation clustering (Azzalini 2007). (A) An example two dimensional dataset. (B) First, the probability density $\hat{f}(y)$ of the point y is estimated by a Gaussian kernel method. The red dots are high-density points and the gray dots are low-density points. The smoothness of the probability density $\hat{f}(y)$ depends on the bandwidth h. (C) Next, the Delaunay triangulation is calculated to evaluate the connection between individual points. (D) Once the probability density $\hat{f}(y)$ and Delaunay triangulation is calculated, the clustering algorithm scans for cluster cores. When the dataset has N points in total, the clustering algorithm scans every N / N_{grid} points in the dataset from the point of maximum probability density. In the k-th scan, the algorithm looks into every point which has higher probability density than the threshold probability density c_p, which is the probability density at the $k\ N_{grid}$-th point from the point of maximum probability density. The points y, which meet $\hat{f}(y) > c_p$, are then analyzed by the connection information based on Delaunay triangulation. In each scan, newly detected points are tested for the following criteria. If the points are not connected to any other existing clusters, the points are labeled as a new cluster (e.g. the lower cluster in the third panel of (D)). If the points are connected with more than two clusters, the points are labeled as "non-core" (e.g. the fifth panel of (D)). This is repeated from the maximum $\hat{f}(y)$ to the minimum $\hat{f}(y)$ to detect cluster cores. (E) The detected cluster cores. The colored dots are points which was allocated to either cluster cores. The gray dots are points which were not allocated as cores. The "non-core" points are allocated in the following procedure. (F) Density estimation is calculated for each cluster cores. (G) The "non-core" point is then allocated to the core which shows the highest density estimation for the point. Note the dots which were gray in the previous panels are colored with the color of allocated cluster core.
