# Supplementary material for: FASTER: an unsupervised fully automated sleep staging method for mice
Source: Genes Cells. 2013 Apr 28;18(6):502–18. doi: 10.1111/gtc.12053 (PMC3712478; doi:10.1111/gtc.12053)
Supplement: Supplementary file 4 [file gtc0018-0502-SD4.pdf]

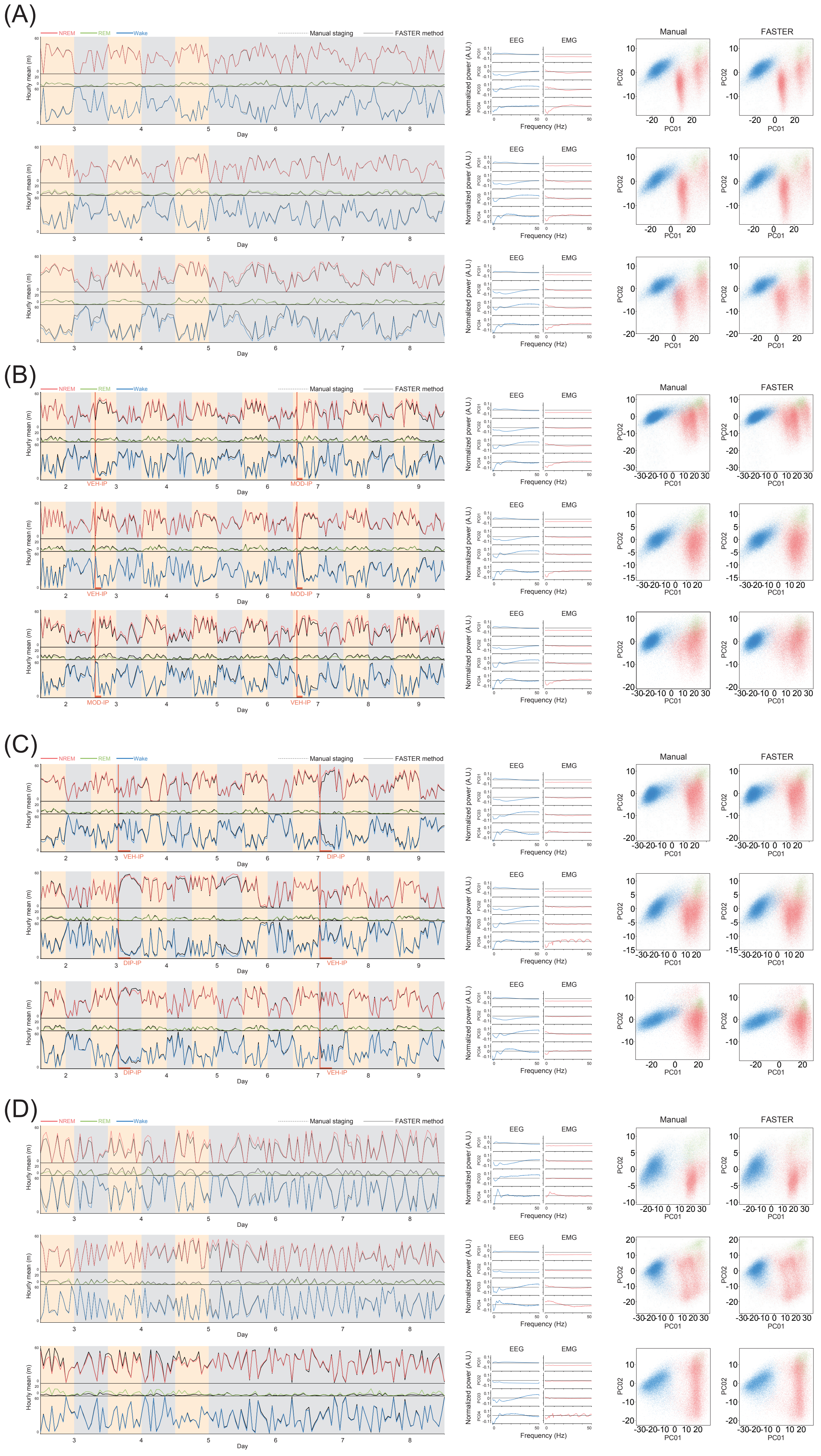

Figure S4 Individual results of FASTER, which were not shown in main figures. Each columns show time series of sleep stages, eigenvectors of top four principal components and scatter plots of first and second principal components colored with manual and automated staging results. (A) Results of staging C57BL/6J mice with FASTER. The circadian rhythm is obvious under constant darkness. (B) Results of staging modafinil induced prolonged wakefulness in C57BL/6J mice with FASTER. (C) Results of staging diphenhydramine induced prolonged sleepiness in C57BL/6J mice with FASTER. (D) Results of staging genetically modified circadian mutant *Bmal1*$^{-/-}$ mice with FASTER. The difference of NREM sleep time between circadian day and night is difficult to detect under constant darkness.
