# Supplementary material for: FASTER: an unsupervised fully automated sleep staging method for mice
Source: Genes Cells. 2013 Apr 28;18(6):502–18. doi: 10.1111/gtc.12053 (PMC3712478; doi:10.1111/gtc.12053)
Supplement: Supplementary file 9 [file gtc0018-0502-SD9.doc]

| Strain | Experiment | Age | Data length | NREM |  |  | REM |  |  | Wake |  |  | Accuracy |
| --- | --- | --- | --- | --- | --- | --- | --- | --- | --- | --- | --- | --- | --- |
|  |  | (weeks) | (days) | Sensitivity | Specificity |  | Sensitivity | Specificity |  | Sensitivity | Specificity |  |  |
| C57BL/6J | Basal | 14.0 | 6 | 94.4% | 96.9% |  | 91.7% | 97.1% |  | 95.6% | 99.0% |  | 94.8% |
| C57BL/6J | Basal | 14.0 | 6 | 96.9% | 97.3% |  | 86.6% | 98.8% |  | 96.6% | 98.9% |  | 96.2% |
| C57BL/6J | Basal | 14.0 | 6 | 95.1% | 97.2% |  | 85.8% | 96.6% |  | 95.1% | 99.2% |  | 94.7% |
| C57BL/6J | Basal | 14.0 | 6 | 98.2% | 90.9% |  | 84.2% | 98.6% |  | 89.3% | 99.6% |  | 92.8% |
| C57BL/6J | MOD-IP | 13.4 | 8 | 94.9% | 86.5% |  | 45.6% | 98.5% |  | 90.3% | 97.1% |  | 89.1% |
| C57BL/6J | MOD-IP | 13.4 | 8 | 97.7% | 92.1% |  | 65.0% | 98.8% |  | 91.8% | 98.8% |  | 92.6% |
| C57BL/6J | MOD-IP | 13.4 | 8 | 97.0% | 94.2% |  | 71.0% | 98.8% |  | 94.6% | 98.3% |  | 93.9% |
| C57BL/6J | DIP-IP | 11.0 | 8 | 94.2% | 94.3% |  | 65.2% | 98.1% |  | 93.4% | 96.6% |  | 92.2% |
| C57BL/6J | DIP-IP | 11.0 | 8 | 97.5% | 93.6% |  | 72.5% | 98.4% |  | 93.1% | 99.4% |  | 94.1% |
| C57BL/6J | DIP-IP | 11.0 | 8 | 96.7% | 93.2% |  | 77.3% | 98.1% |  | 92.0% | 99.3% |  | 93.3% |
| *Bmal1^-/-^* | Basal | 12.0 | 6 | 90.2% | 95.3% |  | 79.5% | 95.9% |  | 95.2% | 98.1% |  | 92.3% |
| *Bmal1^-/-^* | Basal | 12.0 | 6 | 98.2% | 90.2% |  | 44.6% | 99.4% |  | 93.6% | 99.3% |  | 91.7% |
| *Bmal1^-/-^* | Basal | 14.0 | 6 | 97.0% | 91.7% |  | 66.2% | 99.8% |  | 94.1% | 98.0% |  | 93.7% |
