# Supplementary material for: FASTER: an unsupervised fully automated sleep staging method for mice
Source: Genes Cells. 2013 Apr 28;18(6):502–18. doi: 10.1111/gtc.12053 (PMC3712478; doi:10.1111/gtc.12053)
Supplement: Supplementary file 10 [file gtc0018-0502-SD10.doc]

# Supporting Inforamtion/Supplementary Material

**Figure S1** Ratio of eigenvalues of each principal component within the variance of the original signal. The top four components are sufficient to express over 38.4% of the original signal’s variance.

**Figure S2** Optimization results when smoothing factor of density estimation *h_mult_* is selected from 0.2 to 1.4 by 0.2. The panel shows computation time, accuracy, sensitivity and specificity for each stage (from left). In the sensitivity and specificity panel, red, green and blue dots denote NREM sleep, REM sleep and wake, respectively. The points and the shaded area denote mean and standard error of the mean, respectively. Every optimization is done using 5400 epochs randomly from the 6-days-length dataset of four C57BL/6J mice.

**Figure S3** Schematic view of nonparametric density estimation clustering (Azzalini 2007). (A) An example two dimensional dataset. (B) First, the probability density $\hat{f}(y)$ of the point $y$ is estimated by a Gaussian kernel method. The red dots are high-density points and the gray dots are low-density points. The smoothness of the probability density $\hat{f}(y)$ depends on the bandwidth *h*. (C) Next, the Delaunay triangulation is calculated to evaluate the connection between individual points. (D) Once the probability density $\hat{f}(y)$ and Delaunay triangulation is calculated, the clustering algorithm scans for cluster cores. When the dataset has *N* points in total, the clustering algorithm scans every *N / N_grid_* points in the dataset from the point of maximum probability density. In the *k*-th scan, the algorithm looks into every point which has higher probability density than the threshold probability density *c_p_*, which is the probability density at the *k N_grid_* -th point from the point of maximum probability density. The points $y$, which meet $\hat{f}(y)$ > *c_p_*, are then analyzed by the connection information based on Delaunay triangulation. In each scan, newly detected points are tested for the following criteria. If the points are not connected to any other existing clusters, the points are labeled as a new cluster (e.g. the lower cluster in the third panel of (D)). If the points are connected with more than two clusters, the points are labeled as “non-core” (e.g. the fifth panel of (D)). This is repeated from the maximum $\hat{f}(y)$ to the minimum $\hat{f}(y)$ to detect cluster cores. (E) The detected cluster cores. The colored dots are points which was allocated to either cluster cores. The gray dots are points which were not allocated as cores. The “non-core” points are allocated in the following procedure. (F) Density estimation is calculated for each cluster cores. (G) The “non-core” point is then allocated to the core which shows the highest density estimation for the point. Note the dots which were gray in the previous panels are colored with the color of allocated cluster core.

**Figure S4** Individual results of FASTER, which were not shown in main figures. Each columns show time series of sleep stages, eigenvectors of top four principal components and scatter plots of first and second principal components colored with manual and automated staging results. (A) Results of staging C57BL/6J mice with FASTER. The circadian rhythm is obvious under constant darkness. (B) Results of staging modafinil induced prolonged wakefulness in C57BL/6J mice with FASTER. (C) Results of staging diphenhydramine induced prolonged sleepiness in C57BL/6J mice with FASTER. (D) Results of staging genetically modified circadian mutant *Bmal1^-/-^* mice with FASTER. The difference of NREM sleep time between subjective day and night is difficult to detect under constant darkness.

**Table S1** Results from all recordings in this study. Basal: spontaneous sleep-wake cycle under 12:12 h light-dark cycle for the first three days followed by constant darkness for three days; MOD-IP: modafinil IP experiment to induce excessive wakefulness; DIP-IP: diphenhydramine IP experiment to induce excessive sleepiness. See also **Table 1** for summary.

**faster.R** Source code of FASTER written in R (R Core Team 2012). Character extraction, clustering and annotation are implemented. Example one-day-length EEG/EMG data from one of the mice used in optimization of FASTER are included (test.eeg.xdr and test.emg.xdr). The *pdfCluster* package (Azzalini 2012) is necessary to run the code.
